# The Role of Zinc in Bone Tissue Health and Regeneration—a Review

**DOI:** 10.1007/s12011-023-03631-1

**Published:** 2023-04-01

**Authors:** Magda Molenda, Joanna Kolmas

**Affiliations:** Department of Analytical Chemistry and Biomaterials, Faculty of Pharmacy, Medical University of Warsaw, Ul. Banacha 1, 02-097 Warsaw, Poland

**Keywords:** Zinc, Bone tissue, Zinc substitution, Hydroxyapatite, Biomaterials

## Abstract

Zinc is a micronutrient of key importance for human health. An increasing number of studies indicate that zinc plays a significant role in bone tissue’s normal development and maintaining homeostasis. Zinc is not only a component of bone tissue but is also involved in the synthesis of the collagen matrix, mineralization, and bone turnover. It has been demonstrated that zinc can stimulate runt-related transcription factor 2 (Runx2) and promote the differentiation of osteoblasts. On the other hand, zinc has been found to inhibit osteoclast-like cell formation and to decrease bone resorption by stimulating osteoclasts’ apoptosis. Moreover, zinc regulates the RANKL/RANK/OPG pathway, thereby facilitating bone remodeling. To date, not all mechanisms of Zn activity on bone tissue are well understood and documented. The review aimed to present the current state of research on the role of zinc in bone tissue, its beneficial properties, and its effects on bone regeneration. Since calcium phosphates as bone substitute materials are increasingly enriched in zinc ions, the paper included an overview of research on the potential role of such materials in bone filling and regeneration.

## Introduction

Zinc belongs to the group of the most widespread micronutrients. It is considered the most important trace element for human health [1, 2]. It performs not only catalytic or regulatory functions, but also structural ones. In the body of an adult human weighing 70 kg, zinc is stored in the amount of 2 to 2.5 g, mainly in compounds with metallothionein [2, 3]. Fifty percent of this element is found in muscles, 30% in bone tissue, and 20% in other tissues (including testicles, liver, brain, and plasma) [3–5]. Zinc is considered a low-toxic element for humans. The American Food and Nutrition Institute has set the maximum tolerable upper intake level (UL) of zinc for adults at 40 mg/day [6].

Zinc absorption occurs mainly in the small intestine, with greater efficiency from liquids (up to approx. 70%) than from solid foods (approximately 30%) [7]. Depending on its concentration, zinc transport occurs by two mechanisms: passive and facilitated transport, in high and low concentrations, respectively. Importantly, part of the zinc is secreted into the intestines along with pancreatic juice and bile. The absorption of zinc with food also depends on the status of zinc in the body—with its low content, absorption is more efficient [7]. The maintenance of a relatively constant zinc concentration both in the extra- and intracellular spaces is possible due to the presence of specific proteins acting as importers and exporters of this element, regulating the flow of ions into and out of the cell: transporters from the ZnT family (facilitating the diffusion of cations, SLC30) and ZIP (ZRT or Irk-like protein, SLC39) [8]. ZnT proteins reduce the concentration of zinc in the cytoplasm, either transporting zinc outside the cell or moving it to extracellular fluids, while ZIP proteins have the opposite effect—they allow the influx of zinc from the vessels into the cell. Zinc may be transported from the intestinal lumen into the enterocytes via a non-specific divalent metal transporter (DMT1) [7, 9, 10].

Zn is a component or activator of approximately 300 enzymes or their isoforms and therefore significantly impacts the functioning of various human body areas. It is the only metal that is a constituent of all six classes of enzymes [11]. Studies provide that zinc is associated with the activity of about 10% of all proteins in the human body [3]. For example, as a component of zinc-dependent enzymes, i.e., DNA polymerase and thymidine kinase, zinc ensures the proper synthesis of DNA and proteins. In addition, zinc participates in the formation of the correct quaternary structure of many regulatory proteins and hormone receptors, enabling binding to DNA, RNA, or proteins [3, 12]. It produces the so-called zinc fingers, in which the chains of amino acids form a domain (in the shape of a finger) with a centrally located divalent zinc cation, connecting to cysteine, and histidine residues [13–15].

Zinc belongs to the group of the most effective antioxidants—it protects thiol groups of proteins against oxidation [16, 17], and in physiological conditions, it induces metallothioneins with an antioxidant capacity 300 times higher than the ability to capture hydroxyl radicals by glutathione, the most important antioxidant of the cytosol [17].

What is also very important, zinc prevents the excessive production of cyclooxygenase-2 (COX-2), controlling the process of formation of prostaglandins from arachidonic acid. Excess COX-2 leads to enhanced cell proliferation, blocking apoptosis, changes in cell adhesion processes and angiogenesis, and also increases the metastatic capacity of tumor cells, thus contributing to carcinogenesis [18, 19].

A significant role of zinc is also the direct inhibition of some apoptotic enzymes, mainly caspases. Zinc can reduce the level of oxidative stress markers, inhibit the production of C-reactive protein, and block the adhesion of molecules on macrophages and monocytes, protecting the body against inflammatory processes [1, 4].

Therefore, zinc deficiency is one of the major risk factors (ranked 11th by World Health Organization) for morbidity and mortality in developing regions of the world, most dangerous in infants and children [20, 21]. The basic effects of zinc deficiency on the functioning of selected organs are presented in Table 1.

**Table 1:**
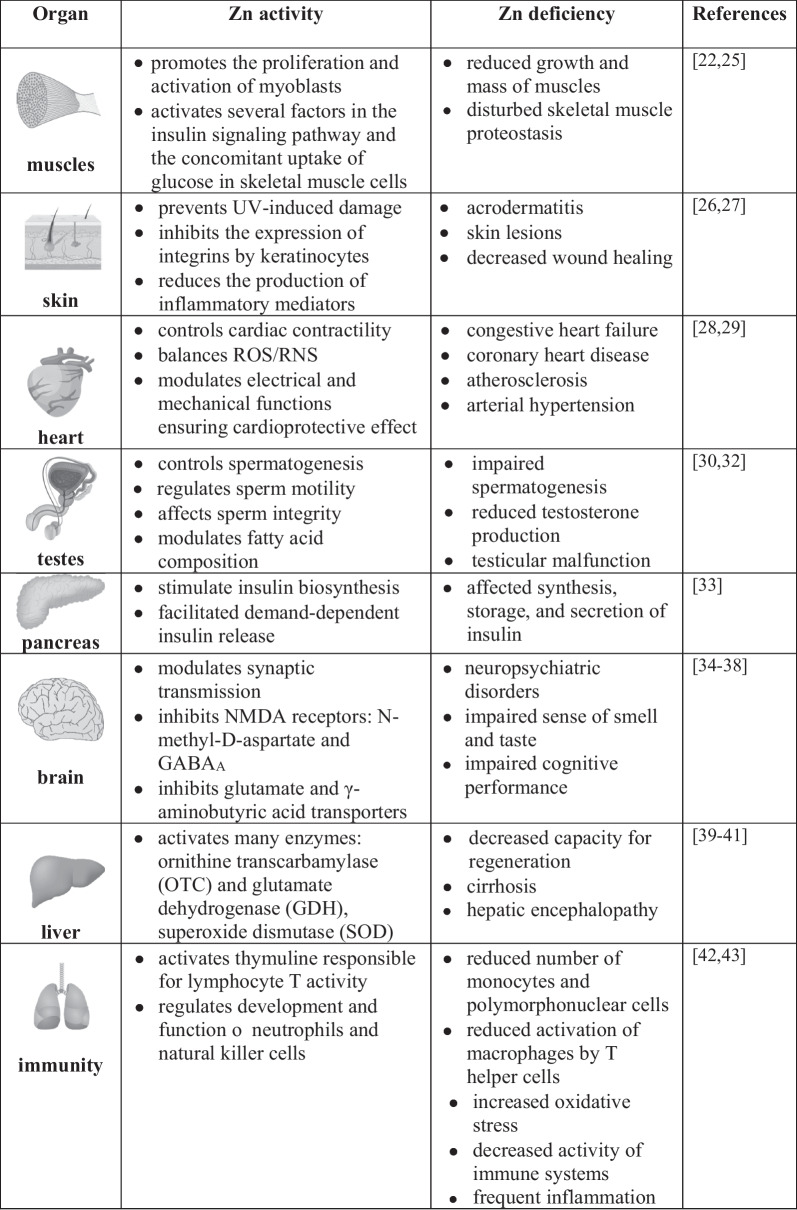
Effect of zinc activity and deficiency in selected human organs

## Zinc and Its Role in Bone Metabolism

Bone tissue is one of the mineralized tissues of the human body. It consists of about 30 wt.% of proteins (mainly collagen type I), 60 wt.% of inorganic compounds (mainly bone apatite), and the rest is water (about 10 wt.%) [44]. Bone tissue is constantly remodeled through a process of coupled bone turnover: briefly, osteoclasts (bone-breaking cells) resorb bone tissue and then osteoblasts fill the resorption sites with new tissue [45].

In addition to calcium and phosphorus, the basic elements that are components of bone apatite, many other macro- and micronutrients affect bone health by participating in the processes of formation or resorption, including interaction with many enzymes involved in them. Zinc belongs to the most important nutrients involved in the metabolism of bone tissue and the proper functioning of the skeletal system [46].

### Effect of Zinc on Bone Metabolism

The effects of zinc supplementation on bone development were discovered over 60 years ago. Prasad et al. noticed decreased levels of zinc in the plasma, erythrocytes, and hair of young boys with dwarfism, hypogonadism, and anemia. The conducted research showed that thegrowth rate of boys with dwarfism was markedly higher in individuals fed with a full-fledged diet supplemented with zinc than in individuals on the same diet without additional amounts of this micronutrient. It was then discovered that nutritional zinc may play a pivotal role in bone development and growth [47].

Zinc in bones, as in other tissues, is a component of many enzymes, but it is also found in the mineral fraction, mainly in bone apatite [48]. Studies conducted on women with osteoporotic disease showed that the zinc content in their bones was lower than in healthy women [49]. Moreover, in studies of postmenopausal women, it was noted that the zinc content in the urine could be an effective macronutrient for bone health (women with osteoporosis excreted over 800 µg of zinc per 1 g of creatinine [49].

In turn, studies conducted on rats and consisting of the supply of various doses of zinc (in the form of zinc sulfate, 5–50 mg Zn /kg of body weight) for 3 days showed not only an increase in the content of zinc in the femur but also DNA, calcium, collagen, and alkaline phosphatase (ALP) [50].

Zinc cations act as cofactors for ALP as well as for collagenase, involved in bone tissue metabolism [51]. ALP belongs to the group of metalloenzymes and contains one magnesium ion and two zinc ions in its active center [52]. Its action is to cleave the phosphate ester bond in compounds such as pyrophosphate, phosphoethanolamine, and pyridoxal phosphate, thereby releasing phosphate ions into the bone matrix, and stimulating its mineralization [52].

Human studies have shown that oral administration of zinc at a dose of 3060 µmol/kg increased both ALP activity as well as the DNA content of the diaphyseal tissues [53].

It is worth noting that the DNA content in the bone is a marker of the number of bone cells: osteoblasts, osteoclasts, and osteocytes [53, 54].

Zinc plays a physiological role in the stimulation of bone growth in cooperation with IGF-I or TGF-β [53, 55]. It may partially interact with tyrosine kinase and tyrosine phosphatase, which are involved in the IGF-I signaling mechanism in cells [56, 57]. Receptors for 1,25-dihydroxy vitamin D3 (calcitriol) have also been shown to have 2 zinc fingers at the DNA binding site. Zinc availability may therefore modulate the effects of calcitriol on bone growth and mineralization [58].

### Zinc in Bone Formation

Studies provided in vitro on osteoblast cells showed that zinc plays a significant role in the process of bone tissue formation [59–61]. Seo et al. showed that zinc treatment of osteoblastic MC3T3-E1 cells affected their proliferation, collagen synthesis, and bone marker protein ALP activity [59].

In vitro studies have been confirmed in an animal model. In studies of oral administration of zinc complexed with beta-Alanyl-L-histidine (beta-alanyl-L-histidine-zinc, AHZ), prolonged administration of significant doses of AHZ in rats stimulate the expression of Runx2/Cbfa1 (core binding factor alpha1), collagen type I, alkaline phosphatase, and osteocalcin (a non-collagenous protein) in cells [62–66].

Runx2/Cbfa1 is a transcription factor, essential for osteoblast differentiation and bone formation. It was found that it serves as a regulatory gene to activate osteoblastogenesis. In turn, the expression of Runx2/Cbfa1 is induced by the activation of BMP-2 signaling. It was established that zinc may induce the BMP-2 signaling pathway and therefore affect osteoblast differentiation [67, 68]. Osterix (osteoblast-specific transcription factor) is also a key regulator of osteogenesis, responsible for preosteoblast differentiation. In addition, Osterix enhances the expression of ZIP1, so that a series of feedback loops occur that will induce zinc influx, osteogenic differentiation, and bone apatite formation [69, 70].

Recent studies have indicated another osteogenic activity of zinc ions [63]. It turns out that zinc ions affect the precipitation and deposition of citrate in bone apatite. It should be noted that citrates are an integral part of osseous apatite. Citrates may facilitate bone mineralization by stabilizing the liquid precursors of calcium phosphates and enhancing their infiltration into the collagen fibrils [63, 70].

In the study provided on ovariectomized rats, it was found that Zn supplementation resulted in a significant increase in ALP activity as well as osteocalcin content. The results obtained in this work confirmed that zinc has a definite effect on osteoblastogenesis, promoting osteoblast differentiation and proliferation [71]. As far as ALP is generally a marker of immature osteoblasts, osteocalcin influences the next stages of osteoblast differentiation [70, 72, 73].

The key role of zinc in bone formation processes has been confirmed by studies conducted on MC3T3-E1 preosteoblasts in vitro culture using zinc-carbon dot complexes [66]. Bifunctional Zn^2+^-doped carbon dots, new nanomaterials were found to have higher osteogenic activity than observed using undoped carbon dots [66]. In the next step, the experiments were continued in vivo on rat’s calvaria, where Zn-doped carbon dots were used as potential osteogenic agents. For comparison, zinc gluconate was used. The obtained results have shown a high capability for bone formation, ALP activation, and long-term stimulation.

**Fig. 1:**
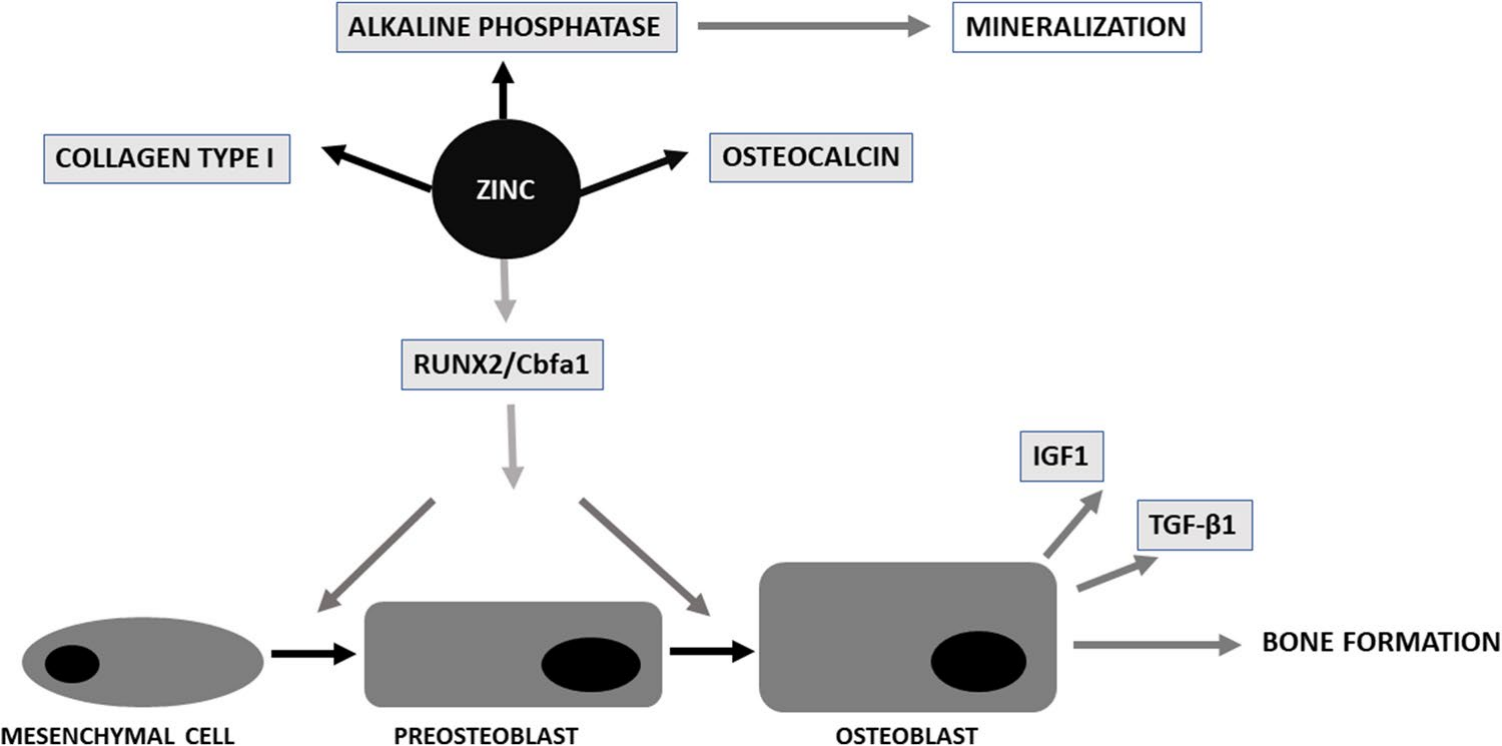
A scheme for Zn activity in bone formation

It has also been shown that zinc can protect osteoblasts from oxidative stress-induced apoptosis by triggering a series of enzyme cascades leading to a decrease in cellular oxidation, inhibition of cytochrome-C release, and a decrease in the phosphorylation of P38 and JNK, involved in cell death signaling. This action of zinc may be used in the prevention of oxidative stress-induced bone diseases such as osteoporosis [54, 70] (Fig. 1).

### Zinc in Bone Resorption

Many years of research on the role of zinc in the metabolism and growth of bone tissue have shown that it also plays a key role in inhibiting bone resorption [54, 62, 74–82].

Moonga and Dempster in the experiments conducted in vitro on isolated rat osteoclasts. The studied cells were extremely sensitive to zinc, even at very low concentrations of zinc ions (10^−14^ M). Moreover, the effect of a significant decrease in bone resorption was specific to zinc and was not observed with other metal ions tested [77].

In a similar experiment, the inhibitory effect of zinc on bone resorption was investigated. The skulls were removed from the rats and then cultured in Dulbecco’s modified Eagle medium for 48 h. The experimental group was treated with different concentrations of AHZ (10^−4^–10^−7^ M). In the PTH control group, prostaglandin E2 and interleukin 1α (bone resorption factors) significantly reduced the calcium content in the examined bone. Interestingly, this effect was not observed in the experimental group. Therefore, it was suggested that zinc may have an inhibitory effect on bone resorption [76].

In [74] it was found that zinc inhibits PTH-stimulated osteoclast-like cell formation mediated by Ca^2+^-dependent protein kinase C. The zinc compound completely inhibits PTH or IL-1α-induced increases in glucose consumption and lactic acid production by bone tissues.

Other studies conducted in an animal model on rats have shown that animals fed food containing no zinc had 50% more osteoclasts in the femoral epiphyseal plate compared to the control group. Zn^2+^ inhibits osteoclastogenesis by decreasing calcineurin phosphatase activity [79, 81].

**Fig. 2:**
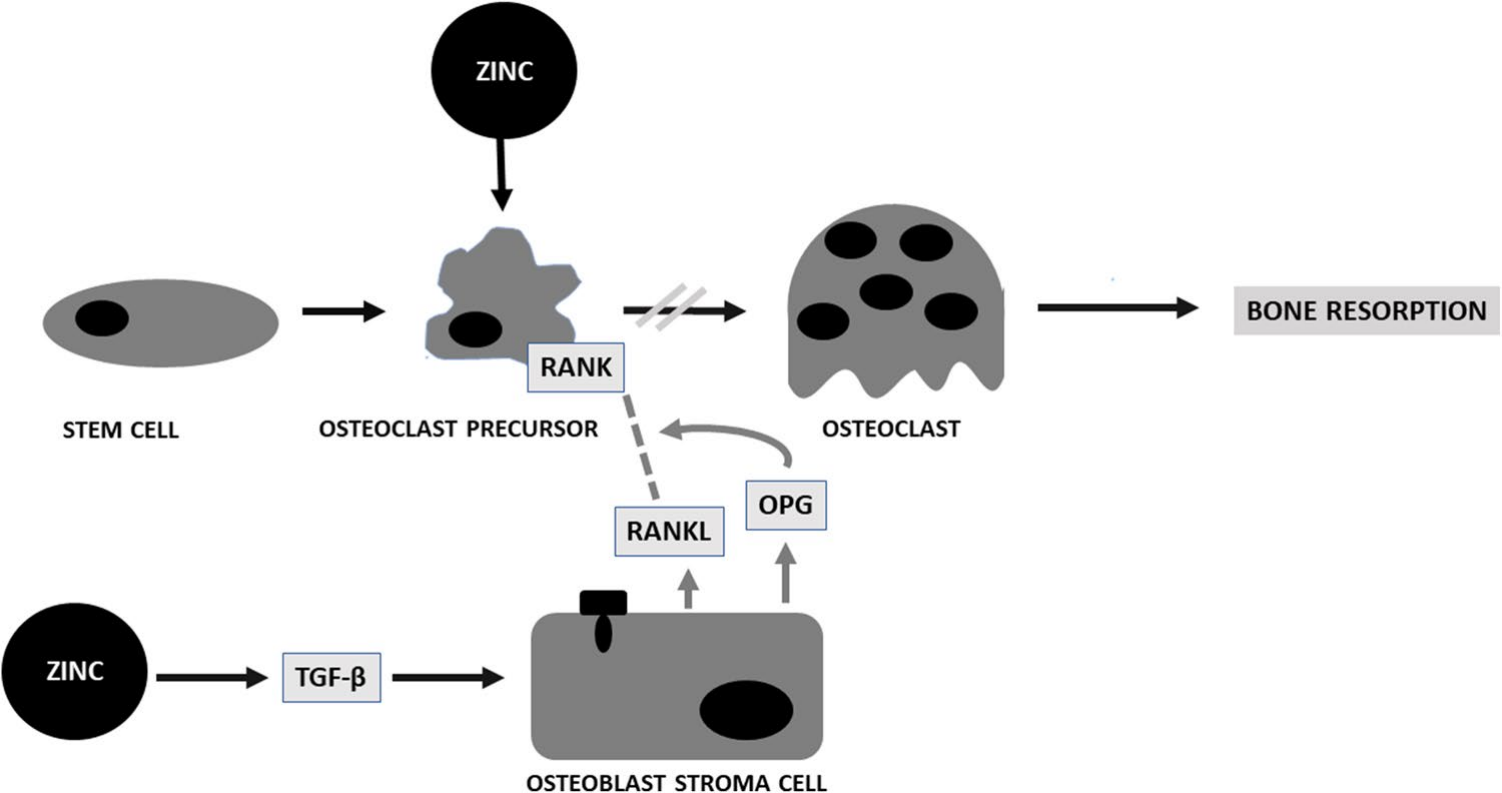
A scheme for Zn activity in bone resorption

Zinc regulates the RANKL/RANK/OPG pathway, thereby facilitating bone remodeling [62, 77–80]. The RANKL/RANK/OPG system is a crucial way for communication between osteoblasts and osteoclasts. It comprises three factors: (1) RANK, a receptor activator of nuclear factor kappa B (NF-κB) expressed on osteoclast precursor cells; (2) RANK ligand (RANKL) found on the surface of osteoblast; and (3) osteoprotegerin (OPG) released by osteoblasts. Zinc was found to inhibit RANKL stimulation as well as signaling pathways associated with it in preosteoclasts [77–80] (see Fig. 2).

Moreover, the available literature contains many references to the inhibitory effect of Zn on TNFα-induced osteoclastogenesis by inhibition of RANKL stimulation in osteoclast precursor cells [80–82].

## Zinc—Antibacterial Activity

Zinc, in addition to its physiological role in the human body, in ionic and nanoparticle forms (particularly in the form of zinc oxide and zinc sulfide) has significant antibacterial activity. It has been shown to exhibit selective toxicity against both Gram-negative and Gram-positive bacteria, with negligible effects on human cells [83, 84].

**Fig. 3:**
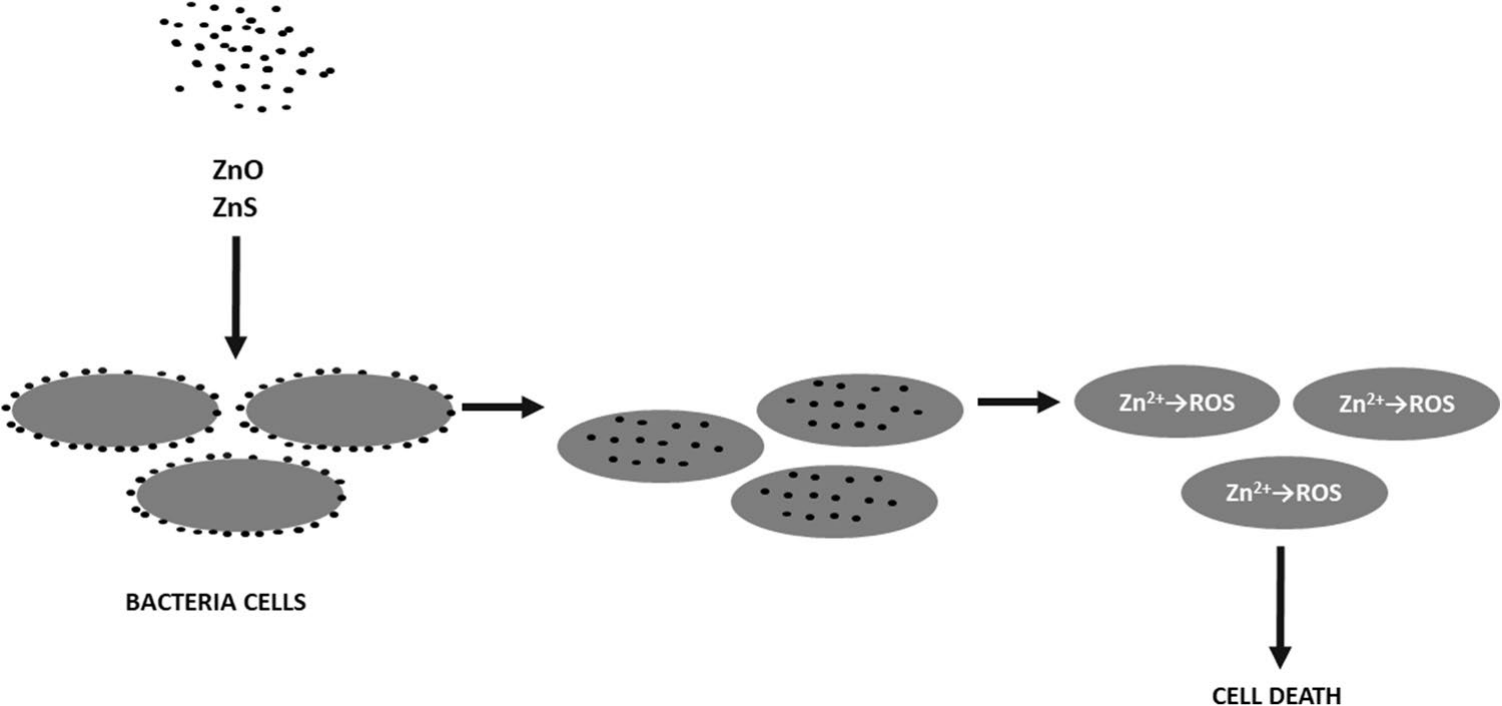
Mechanism of antibacterial effect of nanoparticles of zinc compounds

Zinc oxide nanoparticles (ZnO NP) are some of the most used inorganic materials with bactericidal activity [85]. Due to their high safety in use, they can be found in disinfectants, dental materials, cosmetics, and pharmaceutical preparations. However, it is worth noting that their mechanism of action is not fully understood. It has been shown that the antibacterial activity depends on the dose and size of the nanoparticles—the smaller they are, the greater their toxic effect on microorganisms [86–88]. ZnO and ZnS nanoparticles, under the influence of pH changes and growth factors, move towards and then aggregate on bacterial cells or tumor lesions [89]. As a result of direct contact with the bacterial cell wall, the integrity of the cell membrane is disrupted, the nanoparticles penetrate the cell and release Zn^2+^ ions, the formation of ROS (reactive oxygen species), which induces oxidative stress in the bacterial cell and result initially in cell growth inhibition and then cell death [83, 84, 90, 91] (see Fig. 3).

## Zinc in Bone Replacement Therapy—Calcium Phosphate Biomaterials Containing Zinc Ions

Due to their broad physiological effects, biocompatibility, biodegradability, and pro-regenerative and antibacterial properties, recently metallic biomaterials made of zinc or enriched with zinc (in the form of ions or nanoparticles) are the subject of research by many researchers [83, 85, 87, 92–134]. Of particular interest are biomaterials based on bioceramics calcium phosphate and apatite/polymer composites for the treatment of bone defects in orthopedics and dentistry [100–104, 106–134].

Zinc alloy-based implant materials have gained quite a lot of popularity due to the appropriate time of gradual and H_2_-emission free biodegradation—long enough for local tissues to regenerate completely [93–95]. Zinc alloys are characterized by a medium degree of corrosion and good biocompatibility. Simultaneously, their degradation products—mainly ZnO, Zn(OH)_2_, Zn_3_(PO_4_)_2_ 4H_2_O, are completely bioresorbable [94]. For example, Ca^2+^ ions present in body fluids may react with zinc phosphates and precipitate calcium phosphates (pure or with an admixture of zinc)—compounds with a lower solubility in the aqueous environment [93]. These can then detach from the implants together with the substrate particles and be dissolved or degraded in the physiological environment.

However, zinc alloys also have their disadvantages, including low mechanical strength and the need to produce them by age hardening. Therefore, they are used only as orthopedic fixations (sutures, screws, pins and plates) [94].

Zinc-containing compounds (in the form of nanoparticles or ions) can be used as a coating for conventional metallic implants [95]. The released Zn ions can change the local pH, increasing the alkalinity of the cellular microenvironment and altering the structure of cell transmembrane proteins, which allows cells to bind more easily to proteins adsorbed on the biomaterial surface and promotes adhesion—ensuring proper osteointegration and new bone formation [99]. Using zinc-containing biomaterials accelerates the healing process [92, 93]. Studies have shown that released Zn ions may induce macrophage polarization to a pro-healing phenotype, which facilitates osteogenic differentiation and bone regeneration [94, 95].

Metallic and ceramic materials as well as organic MOFs containing zinc have been described in a review article [85], so the present study focuses on calcium phosphates enriched in zinc ions for potential applications in bone replacement therapy.

According to the available literature, various calcium phosphates with applications in bone replacement therapy are known to have successfully incorporated zinc ions: tricalcium phosphate in β-crystalline form (β-TCP, β-Ca_3_(PO_4_)_2_), brushite (dicalcium phosphate dihydrate, DCPD, CaHPO_4_∙2H_2_O), and monetite (dicalcium phosphate anhydrous, DCPA, CaHPO_4_), and hydroxyapatite (HA, Ca_10_(PO_4_)_6_(OH)_2_) [87, 104, 110, 111].

Due to their greatest chemical similarity to the mineral fraction of bone tissue, hydroxyapatite is among the most interesting. HA is characterized by a high capacity for ionic substitutions, both cationic and anionic [105, 106]. Calcium cations may be partially replaced by other divalent cations (i.e., strontium, magnesium, manganese ions) and other valence ones (i.e., sodium, potassium, gallium, iron ions) [105]. Affinity to substitution depends on the valency of the introduced cations and their radii: cations of the same valency and similar ionic radius can replace calcium cations.

Zinc ions with ionic radii smaller than those of calcium ions (74 vs 100 pm) can be introduced by partial substitution in place of calcium ions or by insertion between two oxygen atoms in a column of OH groups [104].

**Fig. 4:**
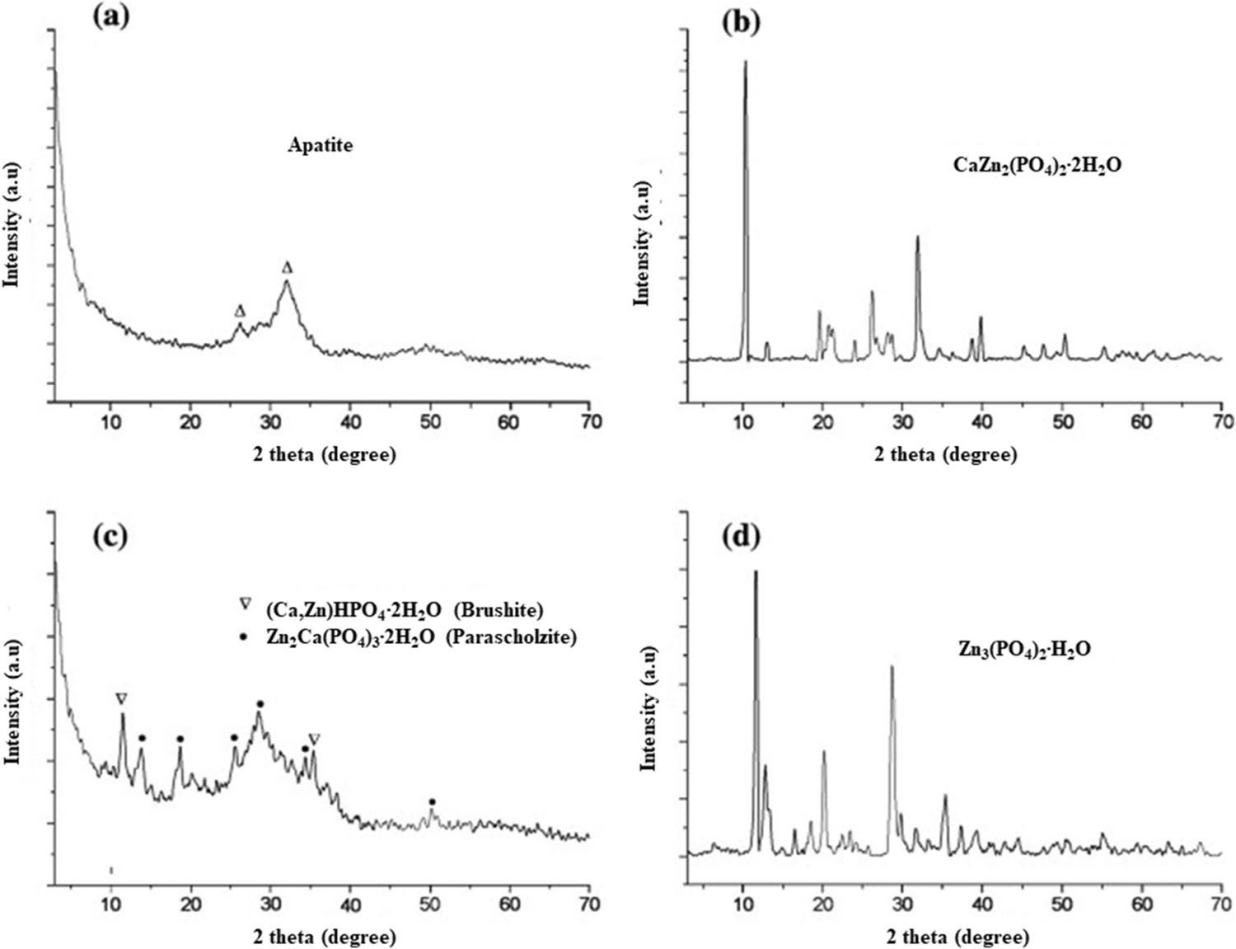
XRD patterns of the samples with Zn fraction over 20 mol. %. **a** Zn40, **b** Zn60, **c** Zn80, and **d** Zn100 according to [108], with permission

Numerous studies on hydroxyapatites have indicated that the limit of zinc introduction is relatively large and, depending on the method of synthesis, can range from 20 to 25 mol% [113, 115, 118]. It is worth noting that above this value, other phases are formed, such as amorphous calcium phosphate with zinc phosphate (see Fig. 4). Importantly, the introduction of zinc ions into the structure of HA alters its physicochemical properties. The crystallite size and the crystallinity index significantly decreased with an increase in Zn concentration [108].

Zinc ions inhibit the crystallization process by altering the lattice parameters of the HA elemental cell, as well as reducing the thermal stability of the material [117, 127, 130].

It appears that at the maximum amount of zinc in hydroxyapatite, it is phase homogeneous up to a temperature of about 700 °C, above which it decomposes with the formation of calcium triphosphate and calcium oxide.

However, such a high concentration of zinc in HA is not necessary to induce a biological response. An in vitro study by Thian et al. [114] indicated that ZnHA with a Zn:(Zn + Ca) ratio of 2.5 mol% had enhanced bioactivity, which is comprised of the ability to form apatite. A significant increase in the growth of human adipose tissue-derived mesenchymal stem cells was observed, along with markers of bone cell differentiation. In addition, bacterial activity tests on this material showed a large decrease in the number of viable *Staphylococcus aureus* bacteria following contact with ZnHA [114].

Zinc present in small amounts in HA is not cytotoxic [128, 133]. HA can enter the cytoplasm through the endosome and decompose into calcium ions and phosphate ions. Stimulation of HMSCs by ZnHA increases the activity of the transcription factor CREB (cAMP response element-binding protein). CREB binds CRE on the promoters of osteogenic genes such as Id1, bone sialoprotein, and osteocalcin, stimulating their transcription. CAMP has also been found to increase the number of hematopoietic stem and progenitor cells (HSPCs).

It is noteworthy that the introduction of zinc ions into hydroxyapatite increases its solubility and bioactivity. The studies presented in [112, 118, 123] showed that this has a beneficial effect on osteoblast proliferation.

In numerous studies of Zn-HA used as metal coatings, its beneficial characteristics such as bioactivity and lack of cytotoxicity have been demonstrated [99, 110, 115]. For example, in a study of titanium implants, where zinc oxide was used as a dopant for HA [99]. Such material had a beneficial effect on increasing osteoblast proliferation and inhibited osteoclast growth. Another study focused on coatings formed from various hydroxyapatites containing Zn, Sr, or Mg ions [110, 115]. The study showed that all materials, including Zn-HA, could improve and accelerate osteointegration. The effect of adding zinc ions to hydroxyapatite has also been studied in animal models. Calasans-Maia et al. used Zn-HA containing 5.0 mol% zinc by introducing it (in powder form or ceramic cylinders) into rat calvaria and rabbits’ tibia bones [132]. In both models, it was shown that the addition of zinc had a beneficial effect on the restoration of bone tissue at the defect site. In contrast, another study [128] examined the effect of ZnHA in apatite/alginate microspheres introduced into damaged rat bone tissue. In vivo studies indicated that: a high accumulation of calcium and zinc in the defect played a key role in inhibiting osteoconduction and thus impaired bone repair.

In the available literature, studies can also be found on other non-apatite calcium phosphates containing zinc ions. Such include zinc-containing calcium phosphate. β-TCP, like hydroxyapatite, has long been used in medicine as a bone substitute biomaterial. Due to its significantly better solubility, it is often combined with HA to improve the bioactivity of the implanted material.

The first studies on the enrichment of β-TCP with zinc ions date back to the 1990s. Bigi et al. [123] obtained zinc-containing TCPs up to 20 mol% at high temperatures (> 1000 °C). Zinc ions, as those having a smaller ionic radius than calcium ions, substitute in their place while affecting changes in the lattice parameters of the TCP elemental cell.

Zinc was found to affect the morphology and mechanical properties of β-TCP by increasing its bulk density. In a study on mouse osteoblasts, Zn-βTCP was shown to stimulate their activity and ALP [125].

In vivo studies of Zn-βTCP-containing materials conducted by Kawamura et al. consistently showed that zinc ions contained even in lesser amounts (5.0 mol%) in calcium phosphate improved rabbit bone tissue regeneration as early as 4 weeks after implantation [124]. On the other hand, the paper [111] presents a study on a canine model indicating an increase in osteoinduction under the influence of zinc ions contained in Zn-βTCP. The good solubility of Zn-βTCP could be used to reveal the antibacterial properties of zinc ions in a shorter period than with Zn-HA. Interestingly, few such studies have been conducted to date.

A calcium phosphate material containing zinc ions is also dicalcium phosphate dihydrate. There is not much work in the available literature exploring its properties and potential applications.

In the work of Laskus-Zakrzewska et al. [104], Zn-DCPD containing different amounts of zinc (ranging from 5 to 20 mol%) were obtained by a standard wet method and their physicochemical properties were investigated in detail. It was found that higher concentrations of zinc ions resulted in the formation of an additional crystalline phase—αZn3(PO4)2, hopeite. At the same time, it was shown that all materials were non-toxic to mouse fibroblasts, which is promising for further research into their potential use, e.g., for the production of docking cement.

An interesting study was presented by Zhao et al. [121]. In this study, they demonstrated the strong antibacterial effect of Zn-DCPD-containing coatings. Simultaneously, they pointed out the anti-corrosive properties of such a coating on an Mg implant.

The presented examples of zinc-containing calcium phosphate materials do not exhaust all possible applications. It is worth mentioning composite materials that replace bone tissue, as well as multifunctional biomaterials that are additionally used as carriers for drugs delivered directly to the bone, where the released zinc ions then act to support the regeneration process.

## Conclusions

The aforementioned studies have indicated that zinc plays a pivotal role in bone remodeling, regeneration, and homeostasis. In vitro and in vivo studies have shown that zinc exhibits multidirectional effects: on the one hand, by promoting osteoblast proliferation and differentiation and protecting osteoblasts from oxidative stress-induced apoptosis; on the other hand, by inhibiting osteoclastogenesis and affecting osteoclast apoptosis. Thanks to its pro-regenerative properties as well as the antibacterial activity, it has been possible to use it in bone substitutes and implant biomaterials with successful results. Based on the presented results, zinc incorporated into various calcium phosphates (hydroxyapatite, β-TCP, brushite, or monetite) may act as a beneficial agent in bone repair.

Certainly, many aspects are not yet known: the optimal amount of zinc introduced into calcium phosphate, the degradation time of calcium phosphate modified in this way, or its long-term biological properties. Nevertheless, the results of the research conducted so far are promising and indicate a potential improvement in the regenerative properties of calcium-phosphate materials.

## Data Availability

All data and other relevant information are available from the corresponding author upon reasonable request.

## References

[1] Prasad AS (2014). Impact of the discovery of human zinc deficiency on health. J Trace Elem Med Biol.

[2] Chasapis CT, Loutsidou AC, Spiliopolou CA, Stefanidou ME (2012). Zinc and human health: an update. Arch Toxicol.

[3] Roohani N, Hurrell R, Kelishadi R, Schulin R (2020). Zinc and its importance for human health: an integrative review. J Res Med Sci.

[4] Uwitonze AM, Ojeh N, Murerehe J (2020). Zinc adequacy is essential for the maintenance of optimal oral health. Nutrients.

[5] Chasapis CT, Ntoupa PSA, Spiliopoluou CA, Stefanidou ME (2020). Recent aspects of the effects of zinc on human health. Arch Toxicol.

[6] Wuehler S, de Romana DL, Haile D, McDonald CM, Brown KH (2022) Reconsidering the tolerable upper levels of zinc intake among infants and young children: a systematic review of the available evidence. Nutrients 14(9):1938 10.3390/nu14091938 PMC9102402 35565906

[7] Huang T, Yan G, Guan M (2020) Zinc homeostasis in bone: zinc transporters and bone diseases. Int J Mol Sci 21. 10.3390/ijms21041236 10.3390/ijms21041236 PMC7072862 32059605

[8] Kambe T, Taylor KM, Fu D (2021). Zinc transporters and their functional integration in mammalian cells. J Biol Chem.

[9] Mońka I, Wiechuła D (2017). Znaczenie cynku dla organizmu ludzkiego w aspekcie suplementacji tego pierwiastka. Ann Academ Med Silesiensis.

[10] Deng Z, Dailey LA, Soukup J, Stonehuerner J, Richards JD, Callaghan KD, Yang F, Ghio AJ (2009). Zinc transport by respiratory epithelial cells and interaction with iron homeostasis. Biometals.

[11] Maret W (2013). Zinc biochemistry: from a single zinc enzyme to a key element of life. Adv Nutr.

[12] Hou R, He Y, Yan G, Hou S, Xie Z, Liao C (2021). Zinc enzymes in medicinal chemistry. Eur J Med Chem.

[13] Kaur K, Gupta R, Saraf SA, Saraf SK (2014). Zinc: the metal of life. Compr Rev Food Sci Food Saf.

[14] Nguyen LH, Tran TT, Truong LTN, Mai HH, Nguyen TT (2020). Overcharging of the zinc ion in the structure of the zinc-finger protein is needed for DNA binding stability. Biochemistry.

[15] Singh JK, van Attikum H (2021). DNA double-strand break repair: Putting zinc fingers on the sore spot. Semin Cell Dev Biol..

[16] Faure P (2003). Protective effects of antioxidant micronutrients (Vitamin E, zinc, and selenium) in type II diabetes mellitus. Clin Chem Lab Med.

[17] Olechnowicz J, Tinkov A, Skalny A, Suliburska J (2018). Zinc status is associated with inflammation, oxidative stress, lipid, and glucose metabolism. J Physiol Sci.

[18] Fong LYY, Jiang LZY, Farber JL (2005). Dietary zinc modulation of COX-2 expression and lingual and esophageal carcinogenesis in rats. J Nat Cancer Inst.

[19] Giacconi R, Costarelli L, Piacenza F, Basso A, Burkle A, Moreno-Villanueva M, Grune T, Weber D, Stuetz W, Gonos ES (2018). Zinc-induced metallothionein in centenarian offspring from a large european population: The MARK-AGE project. J Gerontol A Biol Sci Med Sci.

[20] World Health Organization (2009). Global health risks: mortality and burden of disease attributable to selected major risks.

[21] Caulfield LE, Black RE (2004). Zinc deficiency. Comparative quantification of health risks.

[22] Zhang X, Kutzler L, Killefer J, Novakofski J (2006). Roles of zinc in skeletal muscle cell growth. FASEB J..

[23] Ohashi K, Nagata Y, Wada E, Zammit PS, Shiozuka M, Matsuda R (2015). Zinc promotes proliferation and activation of myogenic cells via the PI3K/Akt and ERK signaling cascade. Exp Cell Res.

[24] Ferdowsi PV, Ng R, Adulsikas J, Sohal SS, Myers S (2020). Zinc modulates several transcription-factor regulated pathways in mouse skeletal muscle cells. Molecules.

[25] Hernández-Camacho JD, Vicente-García C, Parsons D, Navas-Enamorado I (2020). Zinc at the crossroads of exercise and proteostasis Redox. Biol..

[26] Nistor N, Ciontu L, Frasinariu O, Lupu VV, Ignat A, Streanga V (2016). Acrodermatitis Enteropathica. A case report. Medicine (Baltimore).

[27] Ogawa Y, Kinoshita M, Shimada S, Kawamura T (2018) Zinc and skin disorders. Nutrients 10. 10.3390/nu10020199 10.3390/nu10020199 PMC5852775 29439479

[28] Little PJ, Bhattacharaya R, Moreyra AE, Korichneva IL (2010). Zinc and cardiovascular disease. Nutrition.

[29] Choi S, Liu X, Pan Z (2018). Zinc deficiency and cellular oxidative stress: prognostic implications in cardiovascular diseases. Acta Pharmacol Sinica.

[30] Yamaguchi S, Miura C, Kikuchi K, Miura T (2009). Zinc is an essential trace element for spermatogenesis. PNAS.

[31] Omu AE, Al-Azemi MK, Al-Maghrebi A (2015). Molecular basis for the effects of zinc deficiency on spermatogenesis: An experimental study in the Sprague-Dawley rat model. Indian J Urol.

[32] Peng C, Cheng Q, Liu Y (2022). Marginal zinc deficiency in mice increased the number of abnormal sperm and altered the expression level of spermatogenesis-related genes. Biol Trace Elem Res.

[33] Wang M, Phadke M, Packard D, Yadav D, Gorelick F (2020). Zinc: Roles in pancreatic physiology and disease. Pancreatology.

[34] Kawahara M, Tanaka KI, Kato-Negishi M (2018) Zinc, carnosine, and neurodegenerative diseases. Nutrients 10. 10.3390/nu10020147 10.3390/nu10020147 PMC5852723 29382141

[35] Zhang C, Dischler A, Glover K, Qin Y (2022). Neuronal signaling of zinc: from detection and modulation to function. Open Biol.

[36] Krall RF, Moutal A, Phillips MB, Asraf H, Johnson JW, Khanna R, Hershfinkel M, Aizenman E, Tzounopoulos T (2020) Synaptic zinc inhibition of NMDA receptors depends on the association of GluN2A with the zinc transporter ZnT1. Sci Adv 6. 10.1126/sciadv.abb1515 10.1126/sciadv.abb1515 PMC7458442 32937457

[37] Mlyniec K (2021). Interaction between Zinc, GPR39, BDNF, and neuropeptides in depression. Curr Neuropharmacol.

[38] Siwek M, Szewczyk B, Dudek D (2013). Zinc as a marker of affective disorders. Pharmacol Rep.

[39] Cousins RJ (1979). Regulatory aspects of zinc metabolism in liver and intestine. Nutr Rev.

[40] Grungreiff K, Reinhold D, Wedemeyer H (2016). The role of zinc in liver cirrhosis. Ann Hepat.

[41] Hosui A, Kimura E, Abe S et al (2018) Long-term zinc supplementation improves liver function and decreases the risk of developing hepatocellular carcinoma. Nutrients 10(12):1955 10.3390/nu10121955 PMC6316561 30544767

[42] Zhang G, Xue Y, Fu Y, Bao B, Mao M-Y (2022). Zinc deficiency aggravates oxidative stress leading to inflammation and fibrosis in the lung of mice. Biol Trace Elem Res.

[43] Shankar AH, Prasad AS (1998). Zinc and immune function: the biological basis of altered resistance to infection. Am J Clin Nutr.

[44] Feng X (2009). Chemical and biochemical basis of cell-bone matrix interaction in health and disease..

[45] Lefevre E, Farlay D, Bala B, Subtil F (2019). Compositional and mechanical properties of growing cortical bone tissue: a study of the human fibula. Sci Rep.

[46] Rył A, Miazgowski T, Szylińska A (2021). Bone health in aging man: does zinc and cuprum level matter?. Biomolecules.

[47] Prasad AS, Miale A, Farid Z, Sandstead HH, Schulert AR (1963). Zinc metabolism in patients with the syndrome of iron deficiency anemia, hepatosplenomegaly, dwarfism, and hypogonadism. J Lab Clin Med.

[48] Gaffney-Stomberg E (2019). The impact of trace minerals on bone metabolism. Biol Trace Elem Res.

[49] Nielsen FH, Lukaski HC, Johnson LK, Roughead ZK (2011). Reported zinc, but not copper, intakes influence whole-body bone density, mineral content and T score responses to zinc and copper supplementation in healthy postmenopausal women. Br J Nutr.

[50] Giugliano R, Millward DJ (1984). Growth and zinc homeostasis in the severly Zn-deficient rats. Br J Nutr.

[51] Nizet A, Cavalier E, Stenvinkel P, Haarhaus M, Magnusson P (2020). Bone alkaline phosphatase: An important biomarker in chronic kidney disease - mineral and bone disorder. Clin Chim Acta.

[52] Vimalraj S (2020). Alkaline phosphatase: Structure, expression and its function in bone mineralization. Gene.

[53] Yamaguchi M (2007). Role of zinc in bone metabolism and preventive effect on bone disorder. Biomed Res Trace Elem.

[54] Yamaguchi M (2010). Role of nutritional zinc in the prevention of osteoporosis. Mol Cell Biochem.

[55] Dhingra U, Kisenge R, Sudfeld CR (2020). Lower-dose of zinc for childhood diarrhea - a randomized, multicenter trial. N Engl J Med.

[56] Bennasroune A, Mazot P, Boutterin MC, Vigny M (2010). Activation of the orphan receptor tyrosine kinase ALK by zinc. Biochem Biophys Res Commun.

[57] Bellomo E, Massarotti A, Hogstrand C, Maret W (2014). Zinc ions modulate protein tyrosine phosphatase 1B activity. Metallomics.

[58] Wan LY, Zhang YQ, Chen MD, Liu CB, Wu JF (2015). Relationship of structure and function of DNA-binding domain in vitamin D receptor. Molecules.

[59] Seo H-J, Cho Y-E, Kim T, Shin H-I, Kwun I-S (2010). Zinc may increase bone formation through stimulating cell proliferation, alkaline phosphatase activity and collagen synthesis in osteoblastic MC3T3-E1 cells. Nutr Res Pract.

[60] Hashizume M, Yamaguchi M (1993). Stimulatory effect of b-alanyl-L-histidine zinc on cell proliferation is dependent on protein synthesis in osteoblastic MC3T3-E1 cells. Mol Cell Biochem.

[61] Hashizume M, Yamaguchi M (1994). Effect of b-alanyl-L-histidine zinc on differentiation of osteoblastic MC3T3-E1 cells: increases in alkaline phosphatase activity and protein concentration. Mol Cell Biochem.

[62] Amin N, Clarck CCT, Taghizadeh M, Djafarnejad S (2020). Zinc supplements and bone health: The role of the RANKL-RANK axis as a therapeutic target. J Trace Elem Med Biol.

[63] Fu X, Li Y, Huang T (2018). Runx2/Osterix and zinc uptake synergize to orchestrate osteogenic differentiation and citrate containing bone apatite formation. Adv Sci (Weinh).

[64] Fernandes MH, Alves MM, Cebotarenco M, Ribeiro IAC, Grenho L, Gomes PS, Carmezim MJ, Santos CF (2020). Citrate zinc hydroxyapatite nanorods with enhanced cytocompatibility and osteogenesis for bone regeneration. Mater Sci Eng C Mater Biol Appl.

[65] Meng Y, Yang M, Liu X, Yu W, Yang B (2019). Zn^2+^ -carbon dots, a good biocompatibility nanomaterial applied for bio-imaging and inducing osteoblastic differentiation. Nano.

[66] Wang B, Yang M, Liu L (2019). Osteogenic potential of Zn^2+^-passivated carbon dots for bone regeneration in vivo. Biomater Sci.

[67] Cho E-O, Kwun I-S (2018). Zinc upregulates bone-specific transcription factor Runx2 expression via BMP-2 signaling and Smad-1 phosphorylation in osteoblasts. J Nutr Health.

[68] Yang S, Wan D, Wang D, Phimphilai M (2003). In vitro and in vivo synergistic interactions between the Runx2/Cbfa1 transcription factor and bone morphogenetic protein-2 in stimulating osteoblast differentiation. J Bone Miner Res.

[69] Kwun IS, Cho YE, Lomeda RA, Shin HI, Choi JY, Kang YH, Beattie JH (2010). Zinc deficiency suppresses matrix mineralization and retards osteogenesis transiently with catch-up possibly through Runx 2 modulation. Bone.

[70] Liu Q, Li M, Wang S, Xiao Z, Xiong Y, Wang G (2020). Recent advances of osterix transcription factor in osteoblast differentiation and bone formation. Front Cell Dev Biol.

[71] Yamaguchi M, Hashizume M (1994). Effect of beta-alanyl-L-histidine zinc on protein components in osteoblastic MC3T3-El cells: increase in osteocalcin, insulin-like growth factor-I and transforming growth factor-beta. Mol Cell Biochem.

[72] Li B, Liu H, Jia S (2015). Zinc enhances bone metabolism in ovariectomized rats and exerts anabolic osteoblastic/adipocytic marrow effects ex vivo. Biol Trace Elem Res.

[73] Bancroft GN, Sikavitsas VI, van den Dolder J (2002). Fluid flow increases mineralized matrix deposition in 3D perfusion culture of marrow stromal osteoblasts in a dose-dependent manner. Proc Natl Acad Sci U S A.

[74] Yamaguchi M, Hashizume M (1994). Effect of parathyroid hormone and interleukin-1a in osteoblastic MC3T3-E1 cells: interaction with b-alanyl-L-histidine zinc. Peptides.

[75] Moonga BS, Dempster DW (1995). Zinc is a potent inhibitor of osteoclastic bone resorption in vitro. J Bone Miner Res.

[76] Yamaguchi M, Segawa Y, Shimokawa N (1992). Inhibitory effect of β-Alanyl-*L*-Histidinato zinc on bone resorption in tissue culture. Pharmacol.

[77] Suzuki T, Kajita Y, Katsumata S, Matsuzaki H, Suzuki K (2015). Zinc deficiency increases serum concentrations of parathyroid hormone through a decrease in serum calcium and induces bone fragility in rats. J Nutr Sci Vitamin.

[78] Ferreira ECS, Bortolin RH, Freire-Neto FP, Souza KSC, Bezerra JF, Ururahy MAG, Ramos AMO, Himelfarb ST, Abreu BJ, Didone TVN (2017). Zinc supplementation reduces RANKL/OPG ratio and prevents bone architecture alterations in ovariectomized and type 1 diabetic rat. Nutr Res.

[79] Park KH, Park B, Yoon DS, Kwon SH, Shin DM, Lee JW, Lee HG, Shim JH, Park JH, Lee JM (2013). Zinc inhibits osteoclast differentiation by suppression of Ca^2+^-Calcineurin-NFATc1 signaling pathway. Cell Commun Signal.

[80] Suzuki T, Katsumata S, Matsuzaki H, Suzuki K (2016). Dietary zinc supplementation increased TNFalpha and IL1beta-induced RANKL expression, resulting in a decrease in bone mineral density in rats. J Clin Biochem Nutr.

[81] Yamaguchi M (2004). Role of zinc in regulation of osteoclastogenesis. Biomed Res Trace Elem.

[82] Shin JN, Kim I, Lee JS, Koh GY, Lee ZH, Kim H-H (2002). A novel zinc finger protein that inhibits osteoclastogenesis and the function of tumor necrosis factor receptor-associated factor 6. J Biol Chem.

[83] Maleki-Ghaleh H, Siadati MH, Fallah A, Koc B, Kavanlouei M, Khademi-Azandehi P, Moradpur-Tari E, Omidi Y, Barar J, Beygi-Khosrowshahi Y et al (2021) antibacterial and cellular behaviors of novel zinc-doped hydroxyapatite/graphene nanocomposite for bone tissue engineering. Int J Mol Sci 22. 10.3390/ijms22179564 10.3390/ijms22179564 PMC8431478 34502473

[84] Xie Y, He Y, Irwin PL, Jin T, Shi X (2011). Antibacterial activity and mechanism of action of zinc oxide nanoparticles against campylobacter jejuni. Appl Environ Microbiol.

[85] Su Y, Cockerill I, Wang Y, Quin Y-X, Chang L, Zheng D, Zhu D (2019). Zinc-based biomaterials for regeneration and therapy. Trends Biotechnol.

[86] Subramaniam VD, Prasad SV, Banerjee A, Gopinath M, Murugesan R, Marotta F, Sun XF, Pathak S (2019). Health hazards of nanoparticles: understanding the toxicity mechanism of nanosized ZnO in cosmetic products. Drug Chem Toxicol.

[87] Adawy A, Diaz R (2022). Probing the structure, cytocompatibility, and antimicrobial efficacy of silver-, strontium-, and zinc-doped monetite. ACS Appl Bio Mater.

[88] Singh S (2019). Zinc oxide nanoparticles impacts: cytotoxicity, genotoxicity, developmental toxicity, and neurotoxicity. Toxicol Mech Methods.

[89] Krol A, Pomastowski P, Rafinska K, Railean-Plugaru V, Buszewski B (2017). Zinc oxide nanoparticles: Synthesis, antiseptic activity, and toxicity mechanism. Adv Colloid Interface Sci.

[90] de Souza RC, Haberbeck L, Riella H, Ribeiro D, Carciofi B (2019) Antibacterial activity of zinc oxide nanoparticles synthesized by sonochemical process. Braz J Chem Eng 36(2). 10.1590/0104-6632.20190362s20180027

[91] Sirelkhatim A, Mahmud S, Seeni A, Kaus NHM, Ann LC, Bakhori SKM, Hasan H, Mohamad D (2015). Review on zinc oxide nanoparticles: antibacterial activity and toxicity mechanism. Nano-Micro Letters.

[92] Kim SE, Park K (2020). Recent advances of biphasic calcium phosphate bioceramics for bone tissue regeneration. Adv Exp Med Biol.

[93] Kabir H, Munir K, Wen C, Li Y (2021). Recent research and progress of biodegradable zinc alloys and composites for biomedical applications: biomechanical and biocorrosion perspectives. Bioactive Mater.

[94] Li X, Li Y, Peng S, Ye B, Lin W, Hu J (2013). Effect of zinc ions on improving implant fixation in osteoporotic bone. Connect Tissue Res.

[95] Alghamdi HS (2018). Methods to improve osseointegration of dental implants in low quality (Type-IV) bone: an overview. J Funct Biomater.

[96] Oliveira TM, Berti FCB, Gasoto SC, Schneider B, Stimamiglio MA, Berti LF (2021). Calcium phosphate-based bioceramics in the treatment of osteosarcoma: drug delivery composites and magnetic hyperthermia agents. Front Med Technol.

[97] Hamai R, Shirosaki Y, Miyazaki T (2016). Apatite-forming ability of vinyl phosphonic acid-based copolymer in simulated body fluid: effects of phosphate group content. J Mater Sci Mater Med.

[98] Zhang F, Zhou M, Gu W, Shen Z, Ma X, Lu F, Yang X, Zheng Y, Gou Z (2020). Zinc-/copper-substituted dicalcium silicate cement: advanced biomaterials with enhanced osteogenesis and long-term antibacterial properties. J Mater Chem B.

[99] Zhao Q, Yi L, Jiang L, Ma Y, Lin H, Dong J (2019). Surface functionalization of titanium with zinc/strontium-doped titanium dioxide microporous coating via microarc oxidation. Nanomedicine.

[100] Zhang Y, Liu X, Li Z, Zhu S, Yuan X, Cui Z, Yang X, Chu PK, Wu S (2018). Nano Ag/ZnO-incorporated hydroxyapatite composite coatings: highly effective infection prevention and excellent osteointegration. ACS Appl Mater Interfaces.

[101] Song Y, Wu H, Gao Y, Li J, Lin K, Liu B, Lei X, Cheng P, Zhang S, Wang Y (2020). Zinc silicate/nano-hydroxyapatite/collagen scaffolds promote angiogenesis and bone regeneration via the p38 MAPK pathway in activated monocytes. ACS Appl Mater Interfaces.

[102] Gao H, Dai W, Zhao L, Min J, Wang F (2018). The role of zinc and zinc homeostasis in macrophage function. J Immunol Res.

[103] Qu X, Yang H, Yu Z, Jia B, Qiao H, Zheng Y, Dai K (2020). Serum zinc levels and multiple health outcomes: Implications for zinc-based biomaterials. Bioactive Materials.

[104] Laskus-Zakrzewska A, Zgadzaj A, Kolmas J (2021). Synthesis and physicochemical characterization of Zn-doped brushite. Ceram Int.

[105] Šupová M (2015). Substituted hydroxyapatites for biomedical applications: a review. Ceram Int.

[106] Kolmas J, Krukowski S, Laskus A, Jurkitewicz M (2016). Synthetic hydroxyapatite in pharmaceutical applications. Ceram Int.

[107] Cruz R, Calasans-Maia J, Sartoretto S (2018). Does the incorporation of zinc into calcium phosphate improve bone repair? A systematic review. Ceram Int.

[108] Ren F, Xin R, Ge X, Leng Y (2009). Characterization and structural analysis of zinc-substituted hydroxyapatite. Acta Biomater.

[109] Kazimierczak GJ, Kolmas J, Wojcik M, Kolodynska D, Przekora A (2022). Noncytotoxic zinc-doped nanohydroxyapatite -based bone scaffolds with strong bactericidal, bacteriostatic, and antibiofilm activity. Biomater Adv.

[110] Zhu D, Cockerill I, Su Y (2019). Mechanical strength, biodegradation, and in vitro and in vivo biocompatibility of zinc biomaterials. ACS Appl Mater Interfaces.

[111] Luo X, Barbieri D, Davison N (2014). Zinc in calcium phosphate mediate bone induction: in vitro and in vivo model. Acta Biomater.

[112] Ito A, Kawamura H, Otsuka M (2002). Zinc-releasing calcium phosphate for stimulating bone formation. Mater Sci Eng C.

[113] Boanini E, Gazzano M, Nervi C (2019). Strontium and zinc substitution in beta-tricalcium phosphate: X-ray diffraction, solid-state NMR and ATR-FT-IR study. J Func Biomater.

[114] Thian ES, Konishi T, Kawanobe Y (2013). Zinc-substituted hydroxyapatite: a biomaterial with enhanced bioactivity and antibacterial properties. J Mater Sci Mater Med.

[115] Wang X, Ito A, Sogo Y, Li X, Oyane A (2010). Zinc-containing apatite layers on external fixation rods promoting cell activity. Acta Biomater..

[116] Stanić V, Dimitrijević S, Antić-Stanković J, Mitrić M, Jokić B, Plećaš I, Raičević S (2010). Synthesis, characterization and antimicrobial activity of copper and zinc-doped hydroxyapatite nanopowders. Appl Surf Sci.

[117] Bigi A, Foresti E, Gandolfi M, Gazzano M, Roveri N (1995). Inhibiting effect of zinc on hydroxyapatite crystallisation. J Inorg Biochem.

[118] Sogo Y, Ito A, Fukasawa K, Sakurai T, Ichinose N (2004). Zinc containing hydroxyapatite ceramics to promote osteoblastic cell activity. Mater Sci Tech.

[119] Ovesen J, Møller-Madsen B, Thomsen JS, Danscher G, Mosekilde L (2001). The positive effects of zinc on skeletal strength in growing rats. Bone.

[120] Wang T, Zhang J-C, Chen Y, Xiao P-G, Yang M-S (2007). Effect of zinc ion on the osteogenic and adipogenic differentiation of mouse primary bone marrow stromal cells and the adipocytic transdifferentiation of mouse primary osteoblasts. J Trace Elem Med Biol.

[121] Zhao C, Wu H, Hou P, Ni J, Han P, Zhang X (2016). Enhanced corrosion resistance and antibacterial property of Zn doped DCPD coating on biodegradable Mg. Mater Lett.

[122] Fadeeva IV, Goldberg MA, Preobrazhensky II (2021). Improved cytocompatibility and antibacterial properties of zinc-substituted brushite bone cement based on beta-tricalcium phosphate. J Mater Sci Mater Med.

[123] Kawamura H, Ito A, Miyakawa S, Layrolle O, Ojima K, Ichinose N, Tateishi T (2000). Stimulatory effect of zinc-releasing calcium phosphate implant on bone formation in rabbit femora. J Biomed Mater Res.

[124] Bigi A, Foresti E, Gandolfi M, Gazzano M, Roveri N (1997). Isomorphous substitutions in β-tricalcium phosphate: The different effects of zinc and strontium. J Inorg Biochem.

[125] Yamada Y, Ito A, Kojima H (2008). Inhibitory effect of Zn^2+^ in zinc-containing beta-tricalcium phosphate on the resorbing activity of mature osteoclasts. J Biomed Mater Res. Part B.

[126] Grandjean-Laquerrier A, Laquerrier P, Jallot E (2006). Influence of the zinc concentration of sol-gel derived zinc substituted hydroxyapatite on cytokine production by human monocytes in vitro. Biomaterials.

[127] Ito A, Ojima L, Naito H, Ichinose N, Tateishi T (2000). Preparation, solubility, and cytocompatibility of zinc-releasing calcium phosphate ceramics. J Biomed Mater Res.

[128] Martinez-Zelaya VR, Zarranz L, Herrera EZ (2019). In vitro and in vivo evaluations of nanocrystalline Zn-doped carbonated hydroxyapatite/alginate microspheres: zinc and calcium bioavailability and bone regeneration. Int J Nanomedicine.

[129] Shepherd D, Mucal M (2009). Zinc-substituted hydroxyapatite for the inhibition of osteoporosis. Hydroxyapatite (Hap) for Biomedical Applications.

[130] Tang YZ, Chappell HF, Dove MT, Reeder RJ, Lee YJ (2009). Zinc incorporation into hydroxyapatite. Biomaterials.

[131] Hattori Y (2016). Mechanochemical synthesis of zinc-apatitic calcium phosphate and the controlled release for bone tissue engineering. Drug Dev Ind Pharm.

[132] Calasans-Maia M, Fernandes GVO, Rossi AM (2007). Effect of hydroxyapatite and zinc-containing hydroxyapatite on the osseous repair of critical size defect in rat calvaria. Key Eng Mater.

[133] Predoi D (2019). Zinc-doped hydroxyapatite thin films prepared by sol-gel spin coating procedure. Coatings.

[134] Shepherd D (2014). An in vitro study into the effect of zinc-substituted hydroxyapatite on osteoclast number and activity. J Biomed Mater Res A.

